# Impact of Lipoprotein(a) Levels on Perioperative Outcomes in Cardiac Surgery

**DOI:** 10.3390/cells10112829

**Published:** 2021-10-21

**Authors:** Paul Philipp Heinisch, Maks Mihalj, Markus Huber, Joerg C. Schefold, Alexander Hartmann, Michael Walter, Elisabeth Steinhagen-Thiessen, Juerg Schmidli, Frank Stüber, Lorenz Räber, Markus M Luedi

**Affiliations:** 1 Department of Cardiovascular Surgery, Inselspital, Bern University Hospital, 3010 Bern, Switzerland; paulphilipp.heinisch@extern.insel.ch (P.P.H.); maks.mihalj@insel.ch (M.M.); juerg.schmidli@insel.ch (J.S.); 2 Department of Anaesthesiology and Pain Medicine, Inselspital, Bern University Hospital, University of Bern, 3010 Bern, Switzerland; markus.huber@insel.ch (M.H.); frank.stueber@insel.ch (F.S.); 3 Department of Congenital and Pediatric Heart Surgery, German Heart Center Munich, Technische Universität München, 80636 Munich, Germany; 4 Department of Intensive Care Medicine, Inselspital, Bern University Hospital, University of Bern, 3010 Bern, Switzerland; joerg.schefold@insel.ch; 5 Institut für Klinische Chemie und Laboratoriumsmedizin, Universitätsmedizin Rostock, 18057 Rostock, Germany; alexander.hartmann2@med.uni-rostock.de (A.H.); michael.walter@med.uni-rostock.de (M.W.); 6 Department of Endocrinology and Metabolic Medicine, Divison of Lipid Metabolism, Charité–Universitätsmedizin Berlin, 13353 Berlin, Germany; elisabeth.steinhagen-thiessen@med.uni-rostock.de; 7 Department of Cardiology, Inselspital, Bern University Hospital, 3010 Bern, Switzerland; lorenz.raeber@insel.ch

**Keywords:** cardiovascular disease, lipoprotein(a), cardiac surgery, cardiopulmonary bypass

## Abstract

Altered lipid metabolism has been shown to be of major importance in a range of metabolic diseases, with particular importance in cardiovascular disease (CVD). As a key metabolic product, altered lipoprotein(a) (Lp(a)) levels may be associated with adverse clinical outcomes in high-risk cardiovascular patients undergoing cardiac surgery. We aimed to investigate the impact of the important metabolite Lp(a) on complications and clinical outcomes in high-risk patients. A prospective observational cohort study was performed. Data were derived from the Bern Perioperative Biobank (ClinicalTrials.gov NCT04767685), and included 192 adult patients undergoing elective cardiac surgery. Blood samples were collected at 24 h preoperatively, before induction of general anaesthesia, upon weaning from cardiopulmonary bypass (CPB), and the first morning after surgery. Clinical endpoints included stroke, myocardial infarction, and mortality within 30 days after surgery or within 1 year. Patients were grouped according to their preoperative Lp(a) levels: <30 mg/dL (*n* = 121; 63%) or >30 mg/dL (*n =* 71, 37%). The groups with increased vs. normal Lp(a) levels were comparable with regard to preoperative demographics and comorbidities. Median age was 67 years (interquartile range (IQR) 60.0, 73.0), with median body mass index (BMI) of 23.1 kg/m^2^ (23.7, 30.4), and the majority of patients being males (75.5%). Over the observational interval, Lp(a) levels decreased in all types of cardiac surgery after CPB (mean decline of approximately −5 mg/dL). While Lp(a) levels decreased in all patients following CPB, this observation was considerably pronounced in patients undergoing deep hypothermic circulatory arrest (DHCA) (decrease to preoperative Lp(a) levels by −35% (95% CI −68, −1.7), *p* = 0.039). Increased Lp(a) levels were neither associated with increased rates of perioperative stroke or major adverse events in patients undergoing cardiac surgery, nor with overall mortality in the perioperative period, or at one year after surgery. Other than for cohorts in neurology and cardiology, elevated Lp(a) might not be a risk factor for perioperative events in cardiac surgery.

## 1. Introduction

Elevated lipoprotein(a) (Lp(a)) levels have previously been shown to be an independent cardiovascular (CV) risk factor and an acute-phase reactant involved in the repair of tissue injury—most likely via angiogenesis [1,2,3]. Lp(a) levels are genetically determined, and are associated with elevated risk of CV disease and calcified aortic stenosis [4,5,6]. The alteration of circulating Lp(a), which has the ability to modify Lp(a) functions, is an important part of Lp(a) metabolism. There are currently limited therapeutic medications that selectively target increased Lp(a); however, a variety of potential treatments are being investigated [7]. Recently, a therapeutic approach with antisense oligonucleotides (APO(a)LRx) was shown to potently and selectively reduce Lp(a) levels [8].

A recent meta-analysis including over 29,000 patients demonstrated an almost linear positive correlation between Lp(a) levels and CV outcomes [9]. The current evidence suggests that the risk of CV disease is increased by over 40% in patients with Lp(a) levels of 30 mg/dL or greater, compared with patients with levels <15 mg/dL, independently of low-density levels of cholesterol (LDL-C) and other risk factors [10,11].

Originally, a cutoff concentration of 30 mg/dLwas defined, above which the risk of myocardial infarction increases [12,13]. Elevated Lp(a) may also lead to acute destabilisation of pre-existing but quiescent atherosclerotic plaques, which may induce acute myocardial infarction and stroke [9,14,15]. Lp(a) has been proven to be associated with coronary severity and clinical outcomes in patients with various types of coronary artery disease (CAD), such as stable CAD or acute myocardial infarction (AMI) [5,16]. In a large cohort of >6000 patients studied by Xu et al., Lp(a) was identified as a strong risk factor for poor prognosis over the long term, and can aid in risk stratification in patients with triple-vessel disease [17]. 

However, to the best of our knowledge, there are currently no studies reporting the impact of preoperative elevated Lp(a) on perioperative outcomes after coronary artery bypass grafting or valvular and aortic cardiac surgery.

In this prospective study using multiple timed blood samples, we examined the role of Lp(a) changes in patients during and after different types of cardiac surgery, and related the data to clinical complications and outcomes. Given the strong collinearity and complex interplay of cause and effect of Lp(a) and lipids, and current therapeutic approaches to selectively target Lp(a), we specifically focused on Lp(a) only.

## 2. Materials and Methods

### 2.1. Cohort Description 

**Figure 1:**
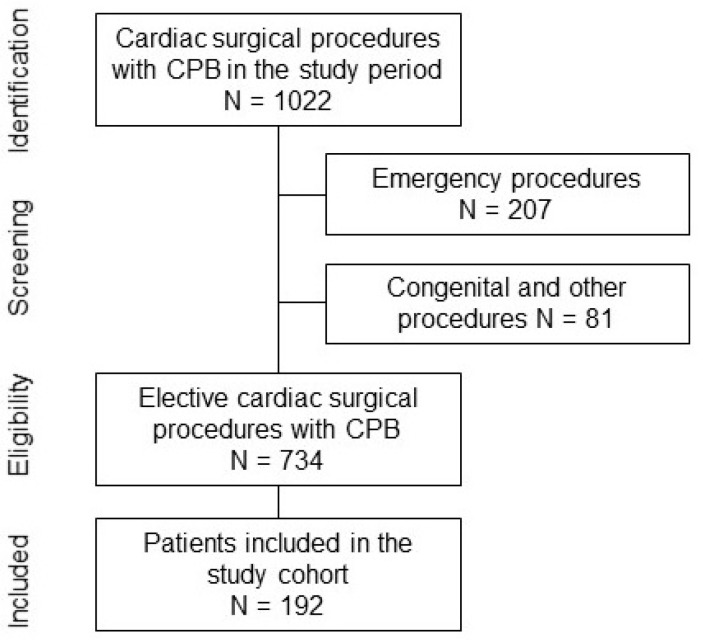
Flow diagram displaying the inclusion of patients. Only non-emergency patients undergoing cardiac surgery with the use of cardiopulmonary bypass (CPB) were included. The inclusion was limited by the operating hours of the institutional biobank, accepting blood samples between 8 a.m. and 4:30 p.m.

This prospective observational cohort study from the Bern Perioperative Biobank (ClinicalTrials.gov NCT04767685) included 192 adult patients who underwent cardiac surgery between January 2019 and December 2019 at our institution (Figure 1). No formal sample size was selected, since this was the first such systematic data collection of Lp(a) and, thus, no reference values were available to estimate possible effect sizes.

Patients were required to provide prior informed consent. Patients who underwent emergent surgeries (defined as <6 h recruitment time between hospital admission and surgery), women with suspected or confirmed pregnancy, and patients who were unable to consent were excluded. Cardiac surgical procedures included coronary artery bypass grafting (CABG) and replacement or repair of the aortic (AVR), mitral (MVR), and tricuspid (TVR) valves, as well as surgery of the ascending aorta or aortic arch. All patients received median sternotomy and cardiopulmonary bypass, either with conventional extracorporeal circulation circuits (CECC) or minimally invasive extracorporeal circulation circuits (MiECC).

### 2.2. Primary and Secondary Outcomes 

The primary outcome was the association of preoperative Lp(a) values (dichotomised according to the threshold of 30 mg/dL) with the incidence of postoperative stroke, 30-day all-cause mortality, and overall survival during the one-year postoperative follow-up period. Secondary endpoints included the association of preoperative Lp(a) values with the postoperative hospitalisation duration, the occurrence of renal complications and the need for renal replacement therapy, the need for postoperative atrial fibrillation (AFIB), the incidence of postoperative myocardial infarction (MI), and the need for re-exploration for bleeding. Peri- and postoperative endpoints were defined according to the guidelines of Akins et al. [18]. The study population, covariates, endpoints, and laboratory analyses are described in the Supplementary Materials. 

Given the different definitions of myocardial infarction amongst guidelines for cardiac surgeons and cardiologists, we manually screened all patient records for clinical signs of acute coronary syndromes during their stay. Specifically, we checked for creatine kinase-MB (CK-MB) levels (3× upper normal limit (UNL)) and respective clinical symptoms with corresponding ST elevation in the ECG, including elevated troponin levels and/or postoperative diagnostic or therapeutic interventions in the cardiac catheterisation lab. Approval by the local ethics committee was obtained for sample collection (KEK Nr. 2018-01272) and for data analysis (KEK Nr. 2019-2000). Ethical guidelines for publishing were adhered to [19].

### 2.3. Collection and Analysis of Blood Samples 

Blood samples (heparin) were collected at 24 h preoperatively (baseline), before induction of general anaesthesia (preoperative), upon weaning from cardiopulmonary bypass (CPB) (intraoperative), and 24 h after surgery (postoperative), and stored at the Bern Liquid Biobank. Biochemical markers were analysed at the Institute for Clinical Chemistry and Laboratory Medicine, Rostock University Medicine, Rostock, Germany. An automated standard biochemical analyser was used to measure the concentrations of relevant indicators, including LDL-C, total cholesterol (TC), high-density lipoprotein cholesterol (HDL-C), and Lp(a). Lp(a) levels were assayed via an immunoturbidimetric method according to the manufacturer’s instructions, using the latex turbidimetric method (LASAY Lp(a) auto; SHIMA Laboratories Co. Ltd., Tokyo, Japan) [20]. Patients were followed up until postoperative day 30, and all-cause mortality was recorded at one year after surgery. 

After recruitment for the Biobank study, all relevant pre-, peri-, and postoperative data for each patient were collected from electronic patient charts (Dendrite Clinical Systems Ltd., Henley on-Thames, UK). Information on all-cause mortality was obtained from the national records or from internal hospital records. Logistic EuroSCORE and EuroSCORE II were calculated to assess the presumed risk of 30-day all-cause mortality. The logistic EuroSCORE and EuroSCORE II range from 0.88 or 0.5, respectively, to <100, representing the percentage risk of perioperative death [21]. A manual check of the clinical records revealed no additional acute coronary syndromes and/or diagnostic or therapeutic interventions in the cardiac catheterisation lab.

### 2.4. Statistical Analysis

Continuous variables were presented as means and standard deviations in the case of normally distributed variables, and as medians and interquartile ranges (IQRs) in other cases. Continuous distributions were examined using the Shapiro–Wilk test for normality and QQ plots. Categorical variables were presented as counts and percentages. The amount and distribution of missing values are provided in the Supplementary Materials. The distribution of preoperative Lp(a) levels and their changes over time are also indicated in the Supplementary Materials.

For categorical variables, group comparisons between patient groups with high preoperative Lp(a) values (defined as above the threshold of 30 mg/dL) and low Lp(a) values (below 30 mg/dL) were based on a permutation chi-squared test (with 2000 permutations) in cases of cell values lower than 5, and on Fisher’s exact test otherwise [13]. For continuous variables, the group comparisons were based on Student’s *t*-test for normally distributed variables, and on the Wilcoxon rank-sum test otherwise. Lp(a) values at different timepoints were presented using geometric means and associated 95% confidence intervals. Bivariate associations of the change in Lp(a) from preoperative to postoperative values for each surgical characteristic (i.e., bypass time) were illustrated using boxplots (in the case of categorical variables) and with locally estimated scatterplot smoothing (LOESS).

As there were multiple primary outcomes, we adjusted the *p*-values using the Benjamini–Hochberg method, and presented both unadjusted and adjusted values. As the secondary analyses were exploratory in nature, no *p*-value adjustment was performed. We further studied the ratio of postoperative to preoperative Lp(a) values, and the association of each surgical characteristic with this ratio was computed with a multivariable linear regression model. For this part of the analysis, we excluded patients whose Lp(a) values were below the measurement threshold (<2 mg/dL), as they all featured the same (default) Lp(a) value (1.98 mg/dL). The fit of the regression model was assessed via examination of the relationship between the model’s residual values and the fitted values. Multicollinearity across the surgical characteristics was studied by computing the variance inflation factor (VIF) for each predictor. For sensitivity analysis, the surgical characteristic with the highest VIF score (bypass time) was excluded from the model, and the corresponding model fit is presented in the Supplementary Materials (Table S2). 

A *p*-value < 0.05 was considered statistically significant. All computations were performed using the R software environment (R version 4.0.2; R Core Team (2020). R: A language and environment for statistical computing; R Foundation for Statistical Computing, Vienna, Austria; URL: https://www.R-project.org/, last accessed on 19 October 2021.

## 3. Results

Patient characteristics, procedural data, and outcomes are shown in Table 1, Table 2, Table 3 and Table 4. Missing data are presented in the Supplementary Material (Table S1).

A total of 192 patients were included in the study (Table 1). Patients were grouped according to their preoperative Lp(a) levels: those with low Lp(a)-levels of <30 mg/dL (*n =* 121; 63%), and those with high Lp(a)-levels of >30 mg/dL (*n =* 71, 37%). Groups were comparable in terms of preoperative demographics and comorbidities, with no significant differences for any variables. The median age was 67 years (interquartile range 60.0, 73.0), with a median body mass index (BMI) of 26.1 kg/m^2^ (23.7, 30.4), and the majority of patients were male (75.5%). The most common comorbidity was arterial hypertension, present in 68.4% of patients, followed by dyslipidaemia (58.1%) and diabetes (18.2%). While levels of preoperative lipoproteins were comparable between groups, the triglycerides were significantly higher in patients with lower Lp(a) levels (1.40 mmol/L (1.04, 1.98) vs. 1.16 mmol/L (0.92, 1.63), *p =* 0.027). Significant carotid disease was present in 7.3% of patients. The median ejection fraction (EF) was 60.0% (55.0, 65.0), and the majority of patients had a low risk of perioperative mortality, with a median EUROSCORE II of 1.73 (0.9, 2.9) and logistic EUROSCORE of 4.65 (2.3, 8.0). The most common procedures included AVR (44.8%) and CABG (40.1%), followed by MVR (23.4%) and ascending aortic replacement (19.8%). Accordingly, the MiECC circuit was used only in isolated CABG cases (22.4%). The two groups had similar CPB and aortic cross-clamp times (104 min (80.0, 132.0) and 68.5 min (52.0, 91.8), respectively), but overall surgery time was significantly longer in the group with higher Lp(a) levels (223 min (188.0, 269.0), vs. 248 min (208.0, 287.0), *p =* 0.030). Of note, we found no statistically difference gender distribution in the low- and high-Lp(a) groups (*p =* 0.461). 

### 3.1. Lp(a) Subanalysis of Primary and Secondary Outcomes

Overall, death occurred in only two patients (1.04%, 95% CI: 0.1–3.7%) within 30 days after surgery (1.65% (95% CI: 0.2–5.8%) in patients with low Lp(a) levels, vs. 0% (95% CI: 0.0–5.1%) in those with high Lp(a) levels, *p* = 0.729), and in only seven patients at 1 year following surgery (4.2% (95% CI: 1.4–9.5%) vs. 2.99% (95% CI: 0.4–10.4%), *p* = 0.729). The finding of no significant association between the Lp(a) group and the primary outcomes was not sensitive with respect to the group threshold of 30 mg/dL (Supplementary Materials).

Stroke rates were comparable between groups (4.2% (95% CI: 1.4–9.5%) in patients with lower Lp(a) levels, vs. 8.45% (95% CI: 3.2–17.5%) in those with higher Lp(a) levels, *p* = 0.729) (Table 2). With respect to all secondary outcomes, we observed no difference in secondary endpoints. Median hospitalisation duration was 7 days in both groups (6.0, 9.0) (*p* = 0.367). The most common complication was postoperative AFIB (24.0% (95% CI: 16.7–32.6%) vs. 21.1% (95% CI: 12.3–32.4%), *p* = 0.784), and postoperative MI occurred in only six patients (3.1%, 95% CI: 0.9–8.3%) (Table 3).

### 3.2. Lp(a) Subanalysis of Pre-, Intra-, and Postoperative Levels Stratified by Perioperative Covariates

**Figure 2:**
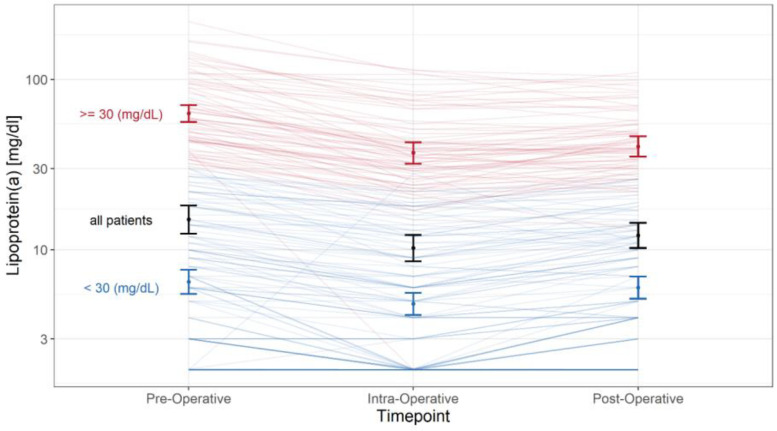
Time series of Lp(a): geometric means and their associated 95% confidence intervals are shown for all patients (in black), high-Lp(a) patients (preoperative Lp(a) ≥30 mg/dL; in red), and low-Lp(a) patients (preoperative Lp(a) <30 mg/dL; in blue).

Lp(a) levels decreased in all types of cardiac surgery after weaning off CPB (median drop of −4 mg/dL (95% CI −7–−1), *p =* 0.004), although this drop was more significant in patients with preoperatively higher levels of Lp(a) of over 30 mg/dL (median drop of −21 mg/dL (95% CI −29–−14), *p* < 0.001) (Figure 2). In all groups, Lp(a) levels had residually recovered by 24 h after surgery; however, a less significant increase was observed in the high-Lp(a) group, but postoperative Lp(a) values in this group were not significantly lower than preoperative values (median difference −4 mg/dL (95% CI −10–2), *p =* 0.172). 

**Figure 3:**
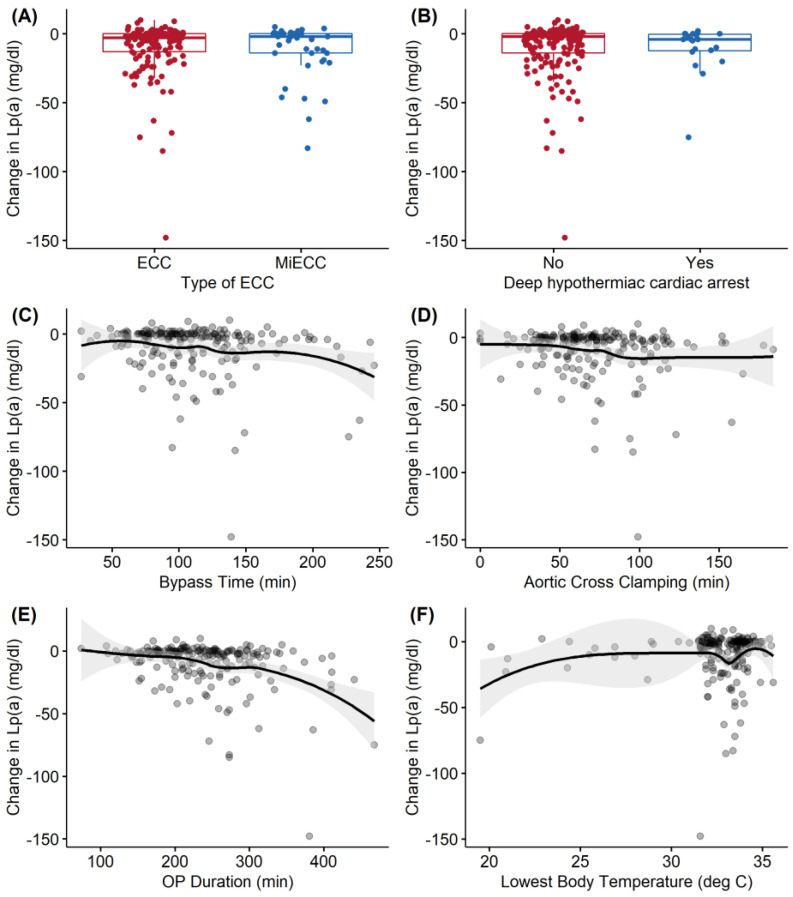
Bivariate associations of surgical characteristics with the change in Lp(a) from preoperative to postoperative values. Boxplots are shown for categorical variables (**A**,**B**), and locally estimated scatterplot smoothing (LOESS) estimates and their associated 95% confidence intervals (grey shading) are depicted for continuous variables (**C**–**F**). Each dot represents the change in Lp(a) for an individual patient.

While Lp(a) levels were decreased in all patients following CPB, this decrease was less consistent in patients with lower Lp(a) levels, and was most evident and significant in patients with deep hypothermic circulatory arrest (DHCA) (decrease in preoperative Lp(a) levels of −35% (95% CI −68, −1.7), *p =* 0.039), followed by operation duration (for each additional hour of surgery, preoperative Lp(a) values decreased by −11% (95% CI −20%, −1.7%), *p =* 0.020) and lower body temperature (for each degree of body temperature increase, the preoperative Lp(a) values decreased by −4.0% (95% CI −8.0%, −0.06%), *p =* 0.046) (Table 4). A sensitivity analysis excluding possible collinear predictors is presented inupplementary Table S3. There were no significant correlations observed between duration of CPB support, aortic cross-clamp time, and perfusion system used (ECC vs. MiECC), as illustrated in Figure 3.

## 4. Discussion

In the present analysis, we observed no influence of Lp(a) levels on perioperative stroke and/or major adverse cardiac and cerebrovascular event (MACCE) rates in patients after coronary, valvular, or aortic surgery. No difference in overall mortality was observed in the perioperative period, nor at one year after surgery.

Perioperative stroke is a severe complication after cardiac surgery, and has the greatest influence of all potential adverse events [14,22]. It has been shown that elevated Lp(a) on admission is an independent risk factor for subsequent adverse cardiovascular events in patients with coronary artery disease [5]. Therefore, laboratory markers to help clinicians identify high-risk patients who might have adverse cardiovascular outcomes seem important.

Lp(a) was proven to be associated with coronary severity and worsened clinical outcomes in patients with different presentations of coronary artery disease [5,6,17]. The structure of apo(a) is very similar to that of plasminogen, the plasminogen’s zymogen, and the primary clot lysis enzyme. Apo(a) inhibits the binding of plasminogen to C-terminal lysines of the cell surface and extracellular matrix proteins. Lp(a) inhibits fibrinolysis and accumulates in the vascular walls of atherosclerotic lesions [8]. In a large cohort study by Xu et al., Lp(a) was identified as an independent predictor of long-term (>6 years of follow-up) adverse clinical outcomes, and might aid in risk stratification in patients with triple-vessel disease [17]. However, thus far, there are no data confirming the influence of preoperative elevated Lp(a) on perioperative outcomes after coronary artery bypass grafting or valvular and aortic cardiac surgery.

The first study reporting on the influence of cardiac surgery on Lp(a) levels included a cohort of only 20 patients [23] receiving isolated CABG, and reported no information on clinical outcomes. To the best of our knowledge, this is the first prospective observational cohort study reporting the impact of Lp(a) levels on clinical outcomes after different types of cardiac surgery. Our study found that Lp(a) levels did not influence primary or secondary outcomes in the early postoperative period. In particular, no difference in perioperative stroke or myocardial infarction was found for different Lp(a) levels.

In the BIOSIGNAL study, Arnold et al. reported on the association between elevated Lp(a) levels and large-artery atherosclerosis stroke. The group concluded that elevated Lp(a) levels were independently associated with stroke aetiology and risk of recurrent cerebrovascular events among primarily Caucasian individuals with evident arteriosclerotic disease [14]. We found no association between different Lp(a) levels and perioperative stroke; however, the incidence of stroke after the perioperative period was not assessed in the current study period.

Some evidence shows that elevated Lp(a) stimulates inflammatory pathways, with respective pathophysiological responses within a few hours [24]. While a study by Cobbaert et al. concluded that Lp(a) levels remain constant during CPB in patients undergoing isolated CABG [23], we found a significant decrease after CPB in patients with higher Lp(a) levels. CPB significantly lowers Lp(a) levels in the initial postoperative period. While Lp(a) levels were decreased in all patients following CPB, this decrease was less consistent in patients with lower Lp(a) levels, and most evident and significant in patients with deep hypothermic circulatory arrest (DHCA) (*p =* 0.039), followed by operation duration (*p =* 0.020) and lower body temperature (*p =* 0.046). No significant correlations were observed between duration of CPB support, aortic cross-clamp time, and perfusion system used (ECC vs. MiECC). Very recently, a trial reported on successful targeting of increased Lp(a) levels [1]. The potential benefits of this option in cardiac surgery remain to be investigated. 

Our study is subject to the limitations of observational research. First, it was a single-centre study, which limits its external validity. Second, the inclusion of patients was restricted by the operating hours of the institutional biobank; however, since no specific policy of patient allocation to the operating room schedule exists at our institution, it seems likely that this would have introduced bias. Third, our study categorised participants according to Lp(a) levels, and did not set distinct cutoff levels, which could have reduced its comparability and clinical applicability. Fourth, CPB may lower Lp(a) levels by the means of dilution, e.g., through the crystalloid or colloid priming volume; however, this effect was likely cancelled out by the last postoperative day. Fifth, only 192 patients were eligible, potentially masking possible effects. Furthermore, the measurement of Lp(a) levels was limited to the perioperative period; therefore, the reliability and scalability of the results we obtained may be limited, and the bivariate relationships illustrated in Figure 2 show some nonlinear behaviour—especially for the lowest body temperature—which warrants caution when examining the results of linear regression. The numbers should be considered as approximations of the underlying nonlinear relationships between these surgical characteristics and the changes in Lp(a) levels. Moreover, it seems important to remember that all DHCA patients have controlled core body temperatures between 19 and 28 degrees Celsius, which was not considered in the multivariable linear regression model. Finally, given the complexity of Lp(a) biology and the enormous challenge of disentangling cause and effect in the biology of Lp(a), HDL, LDL, triglycerides, and other lipids, we focused only on Lp(a). However, this best reflects current therapeutic research to selectively target Lp(a) levels with antisense oligonucleotides [8]. The effects of perioperative lipid status on clinical outcomes after cardiac surgery for the same cohort are described by Mihalj et al. [25]. 

## 5. Conclusions

In summary, increased levels of the key metabolite Lp(a) were not associated with increased perioperative stroke and/or MACCE rates in patients undergoing coronary, valvular, or aortic surgery. The use of CPB was associated with significantly decreased perioperative Lp(a) levels No difference in overall mortality was observed in the early postoperative period, or at one year after surgery, for patients with different Lp(a) levels. 

## Figures and Tables

**Table 1:** Baseline characteristics grouped by preoperative Lp(a).

	All Patients	Low Lp(a) (<30 mg/dL)	High Lp(a) (≥30 mg/dL)	*P*
	*N = 192*	*N = 121*	*N = 71*	
Demographics				
**Age** (years)	67.0 (60.0;73.0)	66.0 (60.0;73.0)	69.0 (61.0;74.5)	0.246
**Height** (cm)	173 (8.71)	173 (8.69)	173 (8.80)	0.593
**Weight** (kg)	80.4 (70.0;90.1)	82.0 (70.7;94.0)	78.4 (70.0;89.4)	0.201
**BMI** (kg/m^2^)	26.1 (23.7;30.4)	26.7 (23.8;30.4)	25.4 (23.4;29.7	0.206
**Sex** (Male)	145 (75.5%)	94 (77.7%)	51 (71.8%)	0.461
Comorbidities				
**Diabetes** (Yes)	35 (18.2%)	25 (20.7%)	10 (14.1%)	0.344
**Diabetes on insulin**				0.667
No	24 (68.6%)	18 (72.0%)	6 (60.0%)	
Yes	11 (31.4%)	7 (28.0%)	4 (40.0%)	
**Hypertension** *^†^* (Yes)	130 (68.4%)	79 (66.4%)	51 (71.8%)	0.535
**Dyslipidaemia** *^†^* (Yes)	111 (58.1%)	70 (58.3%)	41 (57.7%)	1.000
**Nicotine** *^†^*				0.173
Former smoker	49 (26.1%)	36 (30.0%)	13 (19.1%)	
Non-smoker	97 (51.6%)	61 (50.8%)	36 (52.9%)	
Smoker	42 (22.3%)	23 (19.2%)	19 (27.9%)	
**Adipositas** (Yes)	52 (27.1%)	34 (28.1%)	18 (25.4%)	0.806
**Preoperative renal disease** (Yes)	43 (22.4%)	24 (19.8%)	19 (26.8%)	0.351
**Peripheral vascular disease** *^†^*				0.528
No	167 (93.8%)	107 (94.7%)	60 (92.3%)	
Stage 1	4 (2.25%)	3 (2.65%)	1 (1.54%)	
Stage 2	4 (2.25%)	1 (0.88%)	3 (4.62%)	
Stage 3	1 (0.56%)	1 (0.88%)	0 (0.00%)	
Stage 4	2 (1.12%)	1 (0.88%)	1 (1.54%)	
**Carotid disease** *^†^*				0.226
<50%	1 (0.58%)	1 (0.94%)	0 (0.00%)	
>90%	3 (1.75%)	1 (0.94%)	2 (3.08%)	
50–69%,	9 (5.26%)	5 (4.72%)	4 (6.15%)	
70–89%	2 (1.17%)	0 (0.00%)	2 (3.08%)	
no	156 (91.2%)	99 (93.4%)	57 (87.7%)	
**Myocardial infarction** *^†^*				0.832
No MI	171 (89.5%)	106 (88.3%)	65 (91.5%)	
MI 0–7 days before operation	3 (1.57%)	2 (1.67%)	1 (1.41%)	
MI 8–90 days before operation	8 (4.19%)	5 (4.17%)	3 (4.23%)	
MI >90 days before operation	9 (4.71%)	7 (5.83%)	2 (2.82%)	
**COPD** *^†^* (Yes)	23 (12.1%)	17 (14.3%)	6 (8.45%)	0.336
**NYHA** *^†^*				0.879
1	60 (31.4%)	37 (30.8%)	23 (32.4%)	
2	90 (47.1%)	59 (49.2%)	31 (43.7%)	
3	38 (19.9%)	22 (18.3%)	16 (22.5%)	
4	3 (1.57%)	2 (1.67%)	1 (1.41%)	
**CCS** *^†^*				0.950
0	118 (62.4%)	72 (61.0%)	46 (64.8%)	
1	34 (18.0%)	22 (18.6%)	12 (16.9%)	
2	25 (13.2%)	17 (14.4%)	8 (11.3%)	
3	9 (4.76%)	5 (4.24%)	4 (5.63%)	
4	3 (1.59%)	2 (1.69%)	1 (1.41%)	
**Ejection fraction** *^†^*	60.0 (55.0;65.0)	60.0 (55.0;65.0)	60.0 (55.0;65.0)	0.807
**EuroSCORE2** *^†^*	1.73 (0.90;2.93)	1.75 (0.90;2.79)	1.67 (0.91;3.26)	0.924
**Logistic EuroSCORE** *^†^*	4.65 (2.34;8.00)	4.65 (2.44;7.41)	4.92 (2.08;10.9)	0.667
Valve type				
**Aortic valve** (Yes)	86 (44.8%)	55 (45.5%)	31 (43.7%)	0.928
**Mitral valve** (Yes)	45 (23.4%)	30 (24.8%)	15 (21.1%)	0.687
**Tricuspid valve** (Yes)	17 (8.85%)	12 (9.92%)	5 (7.04%)	0.679
**Coronary artery bypass** (Yes)	77 (40.1%)	42 (34.7%)	35 (49.3%)	0.066
**Ascending aortic** (Yes)	38 (19.8%)	23 (19.0%)	15 (21.1%)	0.867
**Aortic arch** (Yes)	11 (5.73%)	7 (5.79%)	4 (5.63%)	1.000
Preoperative Lipoproteins				
**Cholesterol** (mmol/L)	4.42 (1.13)	4.52 (1.15)	4.26 (1.08)	0.123
**HDL cholesterol**(mmol/L)	1.13 (0.92;1.35)	1.08 (0.92;1.36)	1.16 (0.92;1.34)	0.609
**LDL cholesterol** (mmol/L)	2.68 (2.14;3.40)	2.72 (2.30;3.41)	2.47 (2.00;3.26)	0.096
**Quotient overall** (.)	2.50 (1.80;3.20)	2.50 (1.90;3.20)	2.40 (1.60;2.95)	0.149
**Triglycerides** (mmol/L)	1.33 (0.97;1.88)	1.40 (1.04;1.98)	1.16 (0.92;1.63)	0.027
**Lipoprotein(a)** (mg/dl)	15.5 (5.00;45.2)	7.00 (3.00;14.0)	59.0 (44.0;91.5)	< 0.001
Procedural characteristics				
**ECC or MiECC**				0.566
ECC	149 (77.6%)	96 (79.3%)	53 (74.6%)	
MiECC	43 (22.4%)	25 (20.7%)	18 (25.4%)	
**Bypass time** (min)	104 (80.0;132)	103 (78.0;130)	109 (83.0;136)	0.572
**Aortic cross-clamping** (min)	68.5 (52.0;91.8)	68.0 (51.0;90.0)	70.0 (53.5;95.5)	0.774
**Lowest body temperature** (°C)	33.2 (32.1;33.8)	33.3 (32.0;33.9)	33.2 (32.3;33.8)	0.998
**Deep hypothermic cardiac arrest** *^†^* (Yes)	19 (9.95%)	13 (10.7%)	6 (8.57%)	0.816
**Operation duration** (min)	234 (195;276)	223 (188;269)	248 (208;287)	0.030

***^†^*** Includes missing data.

**Table 2:** Primary outcomes.

	All Patients	Low Lp(a) (<30 mg/dL)	High Lp(a) (≥30 mg/dL)	*P*	P-adj *
	*N = 192*	*N = 121*	*N = 71*		
**Postoperative stroke** *^†^*				0.335	0.729
No	179 (94.2%)	114 (95.8%)	65 (91.5%)		
Yes	11 (5.79%)	5 (4.20%)	6 (8.45%)		
**30-day mortality**				0.530	0.729
Died	2 (1.04%)	2 (1.65%)	0 (0.00%)		
Survived	190 (99.0%)	119 (98.3%)	1 (100%)		
**1-Year follow-up** *^‡^*				0.729	0.729
Alive	179 (96.2%)	114 (95.8%)	65 (97.0%)		
Deceased	7 (3.76%)	5 (4.20%)	2 (2.99%)		

*^†^* 2 missing values; *^‡^* 6 missing values; * Benjamini–Hochberg adjustment for multiple comparisons.

**Table 3:** Secondary outcomes.

	All Patients	Low Lp(a) (<30 mg/dL)	High Lp(a) (≥30 mg/dL)	*P*
	*N = 192*	*N = 121*	*N = 71*	
**Length of postoperative hospital stay** (days)	7.00 (6.00;9.00)	7.00 (6.00;9.00)	7.00 (6.00;9.00)	0.367
**Renal complications**				0.370
No	191 (99.5%)	121 (100%)	70 (98.6%)	
Yes	1 (0.52%)	0 (0.00%)	1 (1.41%)	
**Renal replacement therapy**				>0.99
No	191 (99.5%)	120 (99.2%)	71 (100%)	
Yes	1 (0.52%)	1 (0.83%)	0 (0.00%)	
**Postoperative atrial fibrillation**				0.784
No	148 (77.1%)	92 (76.0%)	56 (78.9%)	
Yes	44 (22.9%)	29 (24.0%)	15 (21.1%)	
**Myocardial infarction**				>0.99
No	186 (96.9%)	117 (96.7%)	69 (97.2%)	
Yes	6 (3.12%)	4 (3.31%)	2 (2.82%)	
**Re-exploration for bleeding**				>0.99
No	188 (97.9%)	118 (97.5%)	70 (98.6%)	
Yes	4 (2.08%)	3 (2.48%)	1 (1.41%)	

**Table 4:** Multivariable linear regression of the ratio of postoperative Lp(a) values to preoperative Lp(a) values (%) relative to several surgical characteristics. Note that Lp(a) values below the measurement accuracy (<2 mg/dL) were excluded from the analysis.

	Coefficient	95% CI ^1^	*P*
**ECC or MiECC**			
ECC			
MiECC	9.8	−5.6, 25	0.21
**Bypass time** (hours)	−5.1	−29, 19	0.67
**Aortic cross clamping** (hours)	4.8	−18, 28	0.68
**Lowest body temperature** (°C)	−4.0	−8.0, −0.06	0.046
**Deep hypothermic cardiac arrest**			
No			
Yes	−35	−68, −1.7	0.039
**Operation duration** (hours)	−11	−20, −1.7	0.020

^1^ CI: confidence interval.

## Data Availability

Not available.

## Supplementary Materials

The following are available online at https://www.mdpi.com/article/10.3390/cells10112829/s1, Figure S1: Kernel density distributions of preoperative Lp(a) values (on the log-scale) and their changes; Table S1: Overview of missing data for the analyses; Table S2: Sensitivity analysis of the primary outcomes with regard to the Lp(a) threshold; Table S3: Multivariable linear regression of the ratio of postoperative Lp(a) values to preoperative Lp(a) values.
