# Supplementary material for: Impact of Lipoprotein(a) Levels on Perioperative Outcomes in Cardiac Surgery
Source: Cells. 2021 Oct 21;10(11):2829. doi: 10.3390/cells10112829 (PMC8616553; doi:10.3390/cells10112829)
Supplement: Supplementary file 1 [file cells-10-02829-s001.zip › cells-1368766-supplementary.pdf]

Table S1. Missing data.

Variable	*Number of missing values (% of total)*
**Age** (yr)	0 (0.00%)
**Height** (cm)	0 (0.00%)
**Weight** (kg)	0 (0.00%)
**BMI** (kg/m^2)	0 (0.00%)
**Sex**	0 (0.00%)
**Diabetes**	0 (0.00%)
**Diabetes on insulin**	157 (81.8%)
**Hypertension**	2 (1.04%)
**Dyslipidaemia**	1 (0.52%)
**Nicotine**	4 (2.08%)
**Adipositas**	0 (0.00%)
**Preoperative renal disease**	0 (0.00%)
**Peripheral vascular disease**	14 (7.29%)
**Carotid disease**	21 (10.9%)
**Myocardial infarction**	1 (0.52%)
**COPD**	2 (1.04%)
**NYHA**	1 (0.52%)
**CCS**	3 (1.56%)
**Ejection fraction**	1 (0.52%)
**EuroSCORE2**	8 (4.17%)
**Logistic EuroSCORE**	5 (2.60%)
**Aortic valve**	0 (0.00%)
**Mitral valve**	0 (0.00%)
**Tricuspid valve**	0 (0.00%)
**Coronary Artery Bypass**	0 (0.00%)
**Ascending Aortic**	0 (0.00%)
**Aortic Arch**	0 (0.00%)
**Cholesterol** (mmol/L)	0 (0.00%)
**HDL cholesterol**(mmol/L)	0 (0.00%)
**LDL Cholesterol** (mmol/L)	0 (0.00%)
**Quotient overall** (.)	0 (0.00%)
**Triglycerides** (mmol/L)	0 (0.00%)
**Lipoprotein(a)** (mg/dl)	0 (0.00%)
**ECC or MiECC**	0 (0.00%)
**Bypass time** (min)	0 (0.00%)
**Aortic cross clamping** (min)	0 (0.00%)
**Lowest body temperature** (deg C)	0 (0.00%)
**Deep hypothermic cardiac arrest**	1 (0.52%)
**Operation duration** (min)	0 (0.00%)

Table S2. Sensitivity analysis of the primary outcomes with regard to the Lp(a) threshold. Unadjuasted *P* values are shown.

Threshold value	**15 mg/dL**	**20 mg/dL**	**25 mg/dL**	**30 mg/dL**	**35 mg/dL**	**40 mg/dL**	**45 mg/dL**	**50 mg/dL**
**Postoperative stroke**[†]	0.558	0.773	0.540	0.370	>0.99	>0.99	0.732	>0.99
**30-day mortality**	0.491	0.506	0.522	0.534	0.559	0.584	>0.99	>0.99
**1 Year follow-up**[‡]	0.455	0.468	0.698	0.716	>0.99	0.452	0.690	0.699

Table S3. Multivariable linear regression of the ratio of postoperative Lp(a) values to preoperative Lp(a) values (in percent) on several surgical characteristics. Note that Lp(a) values below the measurement accuracy (< 2 mg/dl) were excluded from the analysis.

	Coefficient	95% CI[1]	*P*
**ECC or MiECC**			
ECC	—	—	
MiECC	11	-3.8, 25	0.15
**Aortic cross clamping** (hours)	0.78	-12, 14	>0.9
**Lowest body temperature** (degrees C)	-3.9	-7.8, 0.02	0.051
**Deep hypothermiac cardiac arrest**			
No	—	—	
Yes	-35	-68, -2.2	0.037
**Operation duration** (hours)	-12	-19, -5.0	<0.001

[1]CI = Confidence Interval.

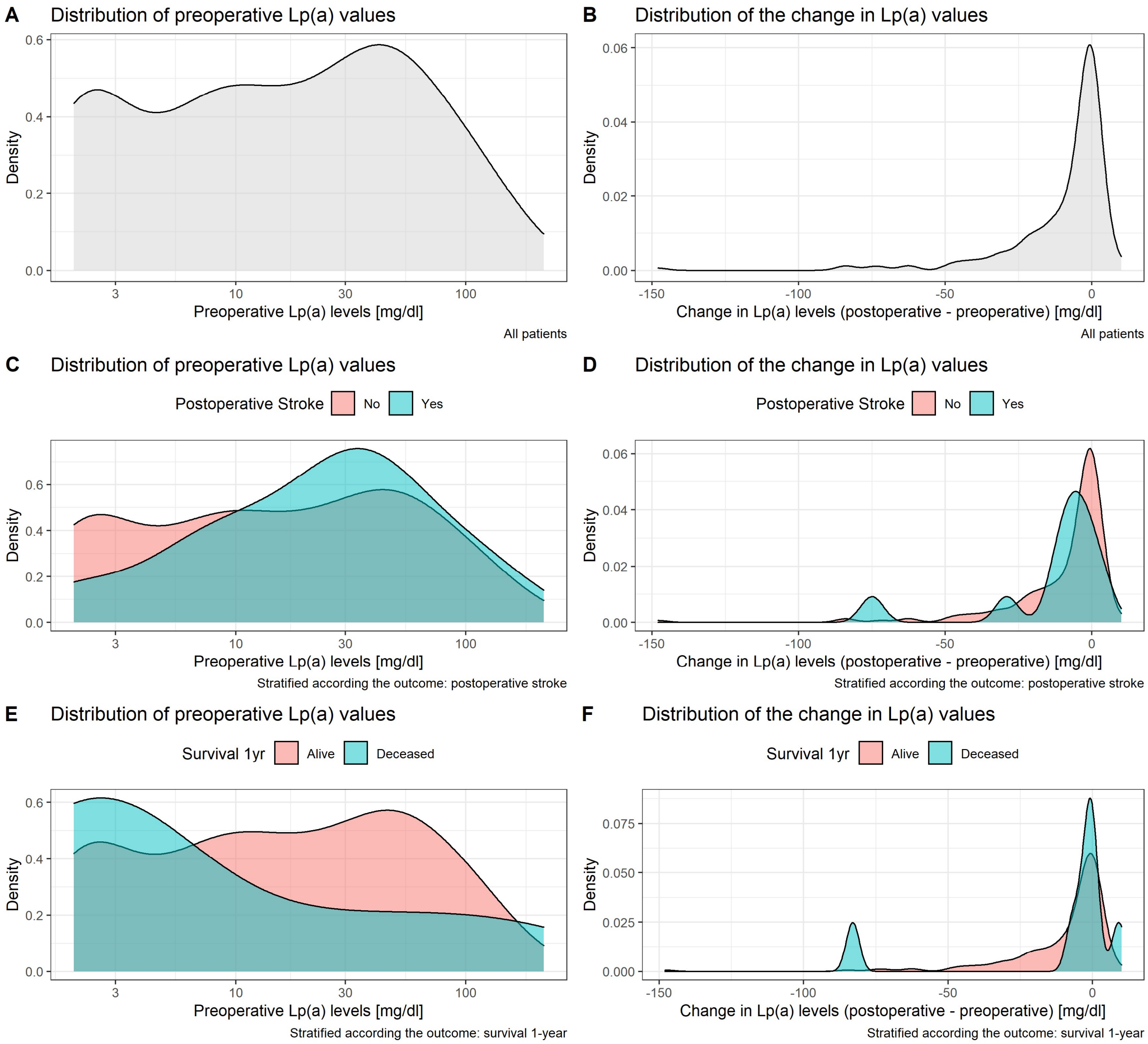

Figure S1. Kernel density distributions of preoperative Lp(a) values (on the log-scale) and their changes (origianl scale) for all patients (panels **A**-**B**), stratified according to the outcome postoperative stroke (**C**-**D**) and stratified according to 1-year survival (**E**-**F**).
